# The association between social anxiety disorder and parenting style among nursing students

**DOI:** 10.1186/s12912-024-02584-7

**Published:** 2024-12-18

**Authors:** Rehab Alhazmi

**Affiliations:** 1 College of Nursing, King Saud bin Abdulaziz University for Health Sciences, Riyadh, Saudi Arabia; 2 King Abdullah International Medical Research Center, Riyadh, Saudi Arabia; 3 Ministry of National Guard Health Affairs, Riyadh, Saudi Arabia

**Keywords:** Social anxiety disorder, Parenting, Nursing students, Saudi Arabia

## Abstract

**Background:**

Social anxiety disorder (SAD) is a common mental health concern affecting students globally and in Saudi Arabia. This study aimed to assess the prevalence of SAD among nursing students and examine its association with various parenting styles.

**Methods:**

This cross-sectional study included 141 female nursing students at the College of Nursing at King Saud bin Abdulaziz University for Health Sciences (KSAU-HS). Participants completed the Parental Authority Questionnaire (PAQ) and Social Phobia Inventory (SPIN). The study data were collected using Microsoft Forms. An ordinal logistic regression analysis was performed to examine the association between SAD and parenting styles.

**Results:**

The prevalence of SAD among the nursing students was 47.5%, with 6.4% having severe SAD and 2.8% having very severe SAD. The authoritative parenting style was the most frequently reported, with 58.2% of the students reporting having an authoritative mother and 44.7% reporting having an authoritative father. Maternal (odds ratio [OR] = 0.181, 95% confidence interval [CI] = 0.062, 0.527, *p* = 0.001) and paternal (OR = 0.186, 95% CI = 0.060, 0.574, *p* = 0.003) authoritative parenting styles were associated with a decrease in the odds of experiencing symptoms of SAD among nursing students. Additionally, a paternal authoritarian parenting style (OR = 2.816, 95% CI = 1.011, 8.218, *p* = 0.048) was associated with an increase in the odds of experiencing SAD symptoms. However, maternal authoritarian parenting style was not significantly associated with SAD symptoms among students (*p* > 0.05).

**Conclusions:**

The parenting style is an influential factor contributing to the experience of SAD in nursing students. In this study, both authoritative and authoritarian parenting styles were significantly associated with the symptoms of SAD. Nursing educators and mental health professionals must establish appropriate strategies to help students cope with SAD. Moreover, educational programs targeting parents must be implemented to increase their awareness of the impact of parental practices on their children’s mental health.

## Background

Social anxiety disorder (SAD) involves experiencing excessive and irrational fear in social settings when performing and being evaluated by others or meeting new people [1]. This intense emotional experience has a detrimental impact on an individual’s daily functioning and fails to reflect the reality of a situation. In Saudi Arabia, SAD is one of the most prevalent psychological disorders, affecting 5.6% of a sample of 4,004 individuals aged between 15 and 65 [2].

SAD is influenced by a variety of factors, including biological, psychological, and environmental factors such as peer relationships and parenting [3]. In recent years, research has increasingly focused on understanding the role of family practices, such as parenting styles, in the development of psychiatric disorders, including SAD [4]. Parents’ behaviors and attitudes strongly influence the development of their children’s thoughts, behaviors, and personalities [5]. Parenting styles comprise the emotions, attitudes, and behaviors of parents toward their children [6]. Baumrind identified three major parenting styles: authoritative, authoritarian, and permissive [5]. Each parenting style has its unique attributes and effects on the psychological well-being of children. Authoritative parenting involves providing encouragement, support, and a moderate level of control to their children [5]. Authoritative parents exhibit affection and responsiveness toward their children while maintaining clear expectations and firm boundaries that their children can adhere to [5]. Children reared in this manner often exhibit higher self-esteem and curiosity than other children [5]. By contrast, authoritarian parents exercise substantial control over their children, enforce strict regulations, and provide fewer opportunities to them [5]. Children of authoritarian parents are expected to comply with their parents’ directions without negotiating, which restricts their capacity to grow and cultivate autonomy [5]. Consequently, they become susceptible to developing low self-esteem, social isolation, and negative thinking patterns [5]. Finally, permissive parents are non-controlling and highly responsive to their children [5]. However, they fail to provide structure or guidance for their children. Children of permissive parents tend to make their own decisions independently, without seeking their parents’ assistance or approval [5]. Consequently, they may experience emotional instability and struggle with self-regulation [5].

Given the impact of parenting styles on psychological health, research has established a relationship between parenting styles and psychiatric disorders, including SAD. In Saudi Arabia, one study demonstrated the negative impact of authoritarian parenting on the development of SAD in adolescents [7]. Several studies conducted worldwide have shown that authoritative parenting has a favorable impact on the psychological well-being of children, whereas authoritarian parenting does not [8, 9]. The adverse effects of parental practices on children may persist well into their old age. Evidence has shown that authoritarian parenting increases the likelihood of anxiety and depression among adults and older adults, whereas authoritative parenting is associated with positive outcomes [10, 11].

While parenting styles influence psychological health, their effect is particularly important for nursing students. Nursing students are exposed to high-stress environments because of the various academic and clinical challenges they face [12]. This stress may be particularly intense in students with SAD. These challenges include struggling with class participation, group projects, language requirements, and interpersonal relationships with peers and instructors, and a fear of receiving low grades [12, 13]. In clinical practice, nursing students may also experience a lack of experience, discomfort from being observed by others, fear of making mistakes and causing harm to patients, exposure to patients dying or suffering, and unfamiliarity with the clinical environment [12, 14]. Cultural and familial expectations may add to the challenges faced by nursing students. In Saudi Arabia, these expectations may include the public’s lack of awareness of the nursing profession and the perception of it as stressful, leading to the profession of nursing being considered unfavorable in society [15]. This public perception of the nursing profession could affect nursing students’ mental health and lead to increased feelings of social anxiety. Family life is also important in Saudi culture [15]. Nursing students may feel pressured to fulfill their family responsibilities while maintaining good academic performance. This can be even more challenging for those raised by authoritarian parents, as they struggle to meet their parents’ expectations and often seek their validation [5]. To alleviate the impact of these factors, nursing institutions must develop programs and strategies to help students, especially those with SAD, cope with these challenges.

Most related research conducted in Saudi Arabia has focused on investigating the occurrence of and factors related to SAD among undergraduate students. Evidence estimates that 16.3–52% of undergraduate students have SAD [16–19]. Among Saudi nursing students, a study showed that 39.8% had symptoms of SAD [20]. Furthermore, factors related to SAD among undergraduate students in Saudi Arabia included younger age, female sex, low income, marital status, parental conflict, birth order, grade point average (GPA), and academic year [16–18]. Studies conducted in Ethiopia, Taiwan, and India found that the prevalence of SAD among undergraduate students ranged from 11.3 to 31.2% [21–23].

Even though SAD is prevalent among students in Saudi Arabia, studies did not address the influence of parenting styles on the development of SAD among nursing students. Therefore, this study aimed to assess the prevalence of SAD among nursing students and examine its association with various parenting styles.

## Methods

### Study design and participants

This was a cross-sectional study with a sample of female undergraduate nursing students at the College of Nursing at King Saud bin Abdulaziz University for Health Sciences (KSAU-HS), Riyadh, Saudi Arabia. The College of Nursing offers a four-year undergraduate nursing program for female students. After successfully completing the program, the students are required to a one-year internship. The target population for this study comprised all students enrolled in the undergraduate nursing program at the time of the study. Those who participated in an internship program were excluded from this study. The data were collected from March 11 to April 30, 2024.

### Sample size and sampling technique

**Fig. 1:**
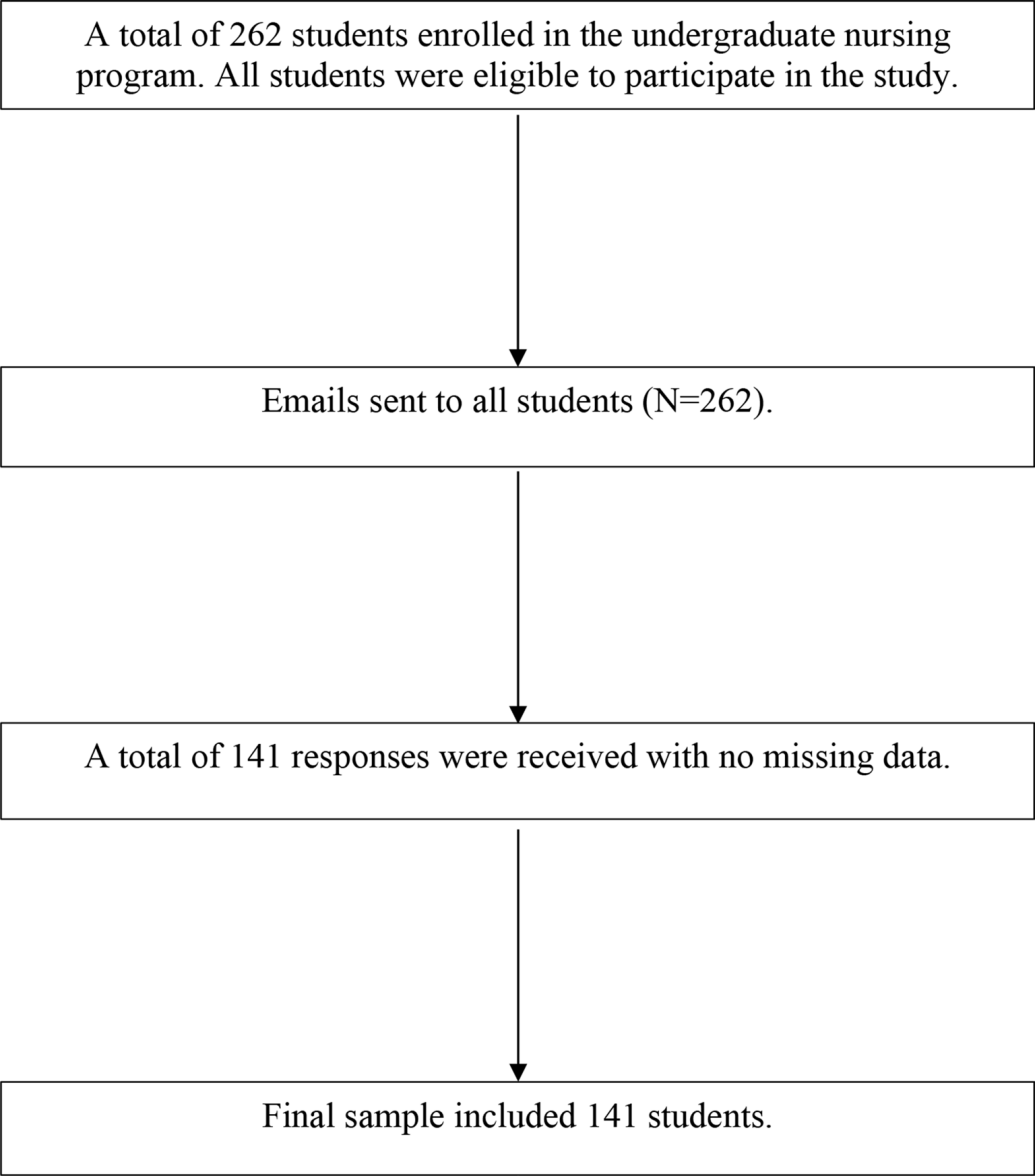
Recruitment process flowchart

Based on the power analysis estimation using G* Power software version 3.1, the required sample size for this study was 74 students. A priori power analysis for logistic regression was performed with a two-tailed test, power level of 0.95, alpha of 0.05, small to medium effect size (OR = 2.67), Pr(Y = 1|X = 1) H0 = 0.45, R2 other X = 0, and normal X distribution with X parm µ as 0 and X parm σ as 1. The effect size estimation was based on previous studies [7, 24]. Additionally, it was interpreted as small (OR = 1.68), medium (OR = 3.47), and large (OR = 6.71), which correspond to Cohen’s d of 0.2, 0.5, and 0.8, respectively [24]. The sample size was also calculated by using the rule of thumb of 10 events per variable [25]. Accordingly, a minimum sample size of 60 is required. Participants were recruited using convenience sampling. Invitation letters, electronic surveys, and consent forms were distributed to 262 students enrolled in the undergraduate nursing program during the study period through their university email addresses. The data were collected via Microsoft Forms. A total of 141 students participated in this study (Fig. 1).

### Measurement of outcomes

The questionnaire was used to collect sociodemographic data, including age, GPA, level of study, birth order, marital status, living arrangement, mother’s education level, father’s education level, parental marital status, family monthly income, and family or personal history of mental illness. The researcher created a sociodemographic questionnaire based on the literature review. The variables of parenting styles and SAD were measured using two established and validated instruments: the Parental Authority Questionnaire (PAQ) and the Social Phobia Inventory (SPIN). The PAQ is a 30-item self-administered questionnaire developed by Buri [26]. It has three subscales: authoritative, authoritarian, and permissive parenting style subscales with 10 items each. The PAQ uses a 5-point Likert scale, with 1 indicating “strongly disagree” and 5 indicating “strongly agree.” Its total scores range from 10 to 50, with higher scores indicating greater use of the corresponding parenting style. Thus, the PAQ is a reliable and valid tool. Its test–retest reliability was 0.77–0.92, and it has adequate internal consistency, with a Cronbach’s alpha ranging from 0.74 to 0.87 [26]. In this study, the Cronbach’s alpha coefficients ranged from 0.68 to 0.81.

The SPIN is a 5-point scale with a 17-item self-administered questionnaire. It was developed by Connor et al. [27] to measure SAD symptoms, including fear, avoidance, and physiological symptoms. The responses range from 0, meaning “not at all,” to 4, meaning “extremely.” The highest possible score on the inventory is 68. The severity of SAD is calculated as follows: 0–20 (none), 21–30 (mild), 31–40 (moderate), 41–50 (severe), and 51–68 (very severe). The SPIN showed good internal consistency, with a Cronbach’s alpha ranging from 0.85 to 0.96 [27]. In this study, the Cronbach’s alpha was 0.92.

### Statistical analysis

Statistical Package for the Social Sciences version 29 was used in this study. Descriptive statistics, including frequencies and percentages, were reported. A chi-square test was conducted to assess the relationship between SAD and sociodemographic variables. An ordinal logistic regression was performed to explore the association between the ordinal outcome variable of SAD and the predictor categorical variable of parenting style (authoritative, authoritarian, and permissive). The assumptions of ordinal logistic regression, including no outliers, independence of observations, no multicollinearity, and proportional odds, were verified. The OR and 95% confidence interval (CI) are reported in the Results section. Post-hoc power analysis was conducted using G* Power software version 3.1. In this study, a significance level of 0.05 was utilized.

### Ethical considerations

This study was approved by the Institutional Review Board (IRB) of the King Abdullah International Medical Research Center (KAIMRC) with approval number (IRB/2125/23). The students provided informed consent before starting the questionnaire. The researcher’s contact information was available on the consent form for participants who required further clarification before participating in the study. Personal identifiable information was not requested or collected from the participants.

## Results

### Sociodemographic characteristics

A total of 141 students completed the online survey and were included in the analysis. Nearly half of the students were 21 years old (48.9%, *n* = 69). Most of the students were in the fourth year of the program (65.2%, *n* = 92) and had a GPA of 3.5–3.99 (34.0%, *n* = 48). Approximately 98% of the students were single (*n* = 139), lived with both parents (83.7%, *n* = 118), and were the middle child (46.8%, *n* = 66). Most students reported no family or personal history of mental illness (72.3%, *n* = 102; Table 1).

**Table 1:** Sociodemographic characteristics of the nursing students (*n* = 141)

Variables		Frequency	Percentage (%)
Age	20	24	17.0
	21	69	48.9
	22	31	22.0
	23	17	12.1
GPA	Less than 3.0	2	1.4
	3.0-3.49	28	19.9
	3.5-3.99	48	34.0
	4 -4.49	38	27.0
	4.50-5.0	25	17.7
Level of study	Third year	49	34.8
	Fourth year	92	65.2
Marital status	Single	139	98.6
	Married	2	1.4
Birth order	Only child	7	5.0
	Oldest child	43	30.5
	Middle child	66	46.8
	Youngest child	25	17.7
Living arrangement	With both parents	118	83.7
	With mother	12	8.5
	With father	8	5.7
	With relatives	1	0.7
	With spouse	2	1.4
Parental marital status	Married and satisfied	84	59.6
	Married and unsatisfied	37	26.2
	Divorced	18	12.8
	Widowed	2	1.4
Mother’s education level	School	37	26.2
	Bachelor	86	61.0
	Master	15	10.6
	Doctoral	3	2.1
Father’s education level	School	32	22.7
	Bachelor	82	58.2
	Master	21	14.9
	Doctoral	6	4.3
Family monthly income	Less than SR 10,000	24	17.0
	SR 10,001-20,000	78	55.3
	More than SR 20,000	39	27.6
Family or personal history of mental illness	Yes	23	16.3
	No	102	72.3
	Don’t know	16	11.3

### Prevalence of SAD and parenting styles

Among the 141 participants, 52.5% reported no SAD symptoms (*n* = 74). Approximately 22% reported mild SAD symptoms (*n* = 31), followed by moderate (16.3%, *n* = 23), severe (6.4%, *n* = 9), and very severe (2.8%, *n* = 4) symptoms of SAD (Table 2).

**Table 2:** Severity of SAD among nursing students (*n* = 141)

Severity of SAD	Frequency	Percentage (%)	
None	74	52.5	
Mild	31	22.0	
Moderate	23	16.3	
Severe	9	6.4	
Very severe	4	2.8	

A total of 82 students reported having authoritative mothers (58.2%) and 63 reported having authoritative fathers (44.7%). Furthermore, 59 students reported having authoritarian fathers (41.8%), 41 reported having authoritarian mothers (29.1%), 19 reported having permissive fathers (13.5%), and 18 reported having permissive mothers (12.8%; Table 3).

**Table 3:** Parenting styles reported by nursing students (*n* = 141)

Parenting style		Frequency	Percentage (%)
Mother	Authoritative	82	58.2
	Authoritarian	41	29.1
	Permissive	18	12.8
Father	Authoritative	63	44.7
	Authoritarian	59	41.8
	Permissive	19	13.5

### Association between sociodemographic characteristics and SAD

Table 4 shows the relationship between SAD and sociodemographic variables. The findings revealed that SAD was not significantly associated with the sociodemographic variables (*p* > 0.05).

**Table 4:** Association between sociodemographic characteristics and SAD (*n* = 141)

Variables		SAD		p-value
		Absent *n* (%)	Present *n* (%)	
Age	20	15 (10.6)	9 (6.3)	0.097
	21	35 (24.8)	34 (24.1)	
	22	16 (11.3)	15 (10.6)	
	23	8 (5.6)	9 (6.3)	
GPA	Less than 3.0	1 (0.7)	1 (0.7)	0.706
	3.0–3.49	16 (11.3)	12 (8.5)	
	3.5–3.99	22 (15.6)	26 (18.4)	
	4–4.49	20 (14.1)	18 (12.7)	
	4.50–5.0	15 (10.6)	10 (7.0)	
Level of study	Third year	29 (20.5)	20 (14.1)	0.248
	Fourth year	45 (31.9)	47 (33.3)	
Marital status	Single	72 (51.0)	67 (47.5)	0.178
	Married	2 (1.4)	0	
Birth order	Only child	3 (2.1)	4 (2.8)	0.854
	Oldest child	23 (16.3)	20 (14.1)	
	Middle child	35 (24.8)	31 (21.9)	
	Youngest child	13 (9.2)	12 (8.5)	
Living arrangement	With both parents	60 (42.5)	58 (41.1)	0.381
	With mother	7 (4.9)	5 (3.5)	
	With father	5 (3.5)	3 (2.1)	
	With relatives	0	1 (0.7)	
	With spouse	2 (1.4)	0	
Parental marital status	Married and satisfied	45 (31.9)	39 (27.6)	0.644
	Married and unsatisfied	17 (12.0)	20 (14.1)	
	Divorced	10 (7.0)	8 (5.6)	
	Widowed	2 (1.4)	0	
Mother’s education level	School	20 (14.1)	17 (12.0)	0.707
	Bachelor	47 (33.3)	39 (27.6)	
	Master	6 (4.2)	9 (6.3)	
	Doctoral	1 (0.7)	2 (1.4)	
Father’s education level	School	18 (12.7)	14 (9.9)	0.169
	Bachelor	38 (26.9)	44 (31.2)	
	Master	13 (9.2)	8 (5.6)	
	Doctoral	5 (3.5)	1 (0.7)	
Family monthly income	Less than SR 10,000	10 (7.0)	14 (9.9)	0.095
	SR 10,001–20,000	44 (31.2)	34 (24.1)	
	More than SR 20,000	20 (14.1)	19 (13.4)	
Family or personal history of mental illness	Yes	17 (12.0)	6 (4.2)	0.152
	No	48 (34.0)	54 (38.2)	
	Don’t know	9 (6.3)	7 (4.9)	

*p* <0.05

### Association between parenting styles and SAD

An ordinal logistic regression was performed to explore the association between the outcome ordinal variable of SAD and the predictor variable of parenting styles, including authoritative, authoritarian, and permissive styles. Maternal authoritative, paternal authoritative, and paternal authoritarian parenting styles were significantly associated with SAD among nursing students. Maternal (OR = 0.181, 95% CI = 0.062, 0.527, *p* = 0.001) and paternal (OR = 0.186, 95% CI = 0.060, 0.574, *p* = 0.003) authoritative parenting styles were associated with a decrease in the odds of experiencing symptoms of SAD among nursing students. However, a paternal authoritarian parenting style (OR = 2.816, 95% CI = 1.011, 8.218, *p* = 0.048) was associated with an increase in the odds of experiencing SAD symptoms. Furthermore, a maternal authoritarian parenting style was not significantly associated with SAD symptoms (*p* > 0.05; Table 5).

**Table 5:** Ordinal logistic regression analysis for the association between parenting styles and SAD (*n* = 141)

Variables		OR	95% CI		*p*-value
			Lower	Upper	
Maternal parenting style	Authoritative	0.181	0.062	0.527	0.001
	Authoritarian	0.808	0.274	2.383	0.690
	Permissive	1			
Paternal parenting style	Authoritative	0.186	0.060	0.574	0.003
	Authoritarian	2.816	1.011	8.218	0.048
	Permissive	1			

*p* < 0.05;1: reference category

### Post-hoc power analyses

Post-hoc power calculations revealed that the study had sufficient power to detect small to medium effect size for paternal authoritarian parenting style (OR = 2.816, 0.99 power). However, it lacked adequate power to detect small effect sizes to include those for paternal authoritative parenting style (OR = 0.186, 0.13 power) and maternal authoritative parenting style (OR = 0.181, 0.13 power).

## Discussion

This study assessed the association between SAD and parenting styles among nursing students at the College of Nursing at KSAU-HS in Riyadh, Saudi Arabia. To the best of the author’s knowledge, no such study has been conducted with nursing students in Saudi Arabia. Therefore, this study contributes to the current literature on the potential variables affecting nursing students’ psychological well-being in Saudi Arabia. The findings demonstrated that 47.5% of the nursing students had symptoms of SAD. This finding is consistent with those of other studies conducted with medical students in Saudi Arabia [16, 17]. For example, a cross-sectional study of 5,896 medical students found significant levels of SAD among participants (51%) [16]. The prevalence in this study was also higher than the reported prevalence of 39.8 in a sample of 138 nursing students in Saudi Arabia [20]. Similarly, this study found a higher prevalence of social anxiety than that identified in two previous studies with students enrolled in different programs in Saudi Arabia [18, 19]. Moreover, the present study found a greater prevalence of SAD than in Ethiopia (31.2%), Taiwan (23.7%), and India (11.3%) [21–23]. The increased prevalence of SAD among female nursing students could be explained by sex differences in vulnerability to anxiety. Females are more biologically and psychologically vulnerable to developing anxiety than males [16]. It could also be attributed to the variations in academic programs and the stressors encountered by students during their education. The nursing program is rigorous, and its clinical components are more stressful than those of other programs [12]. Moreover, cultural differences could provide a potential explanation for the variations between this study and those conducted in other countries. A practical explanation for our findings could be a lack of public awareness of the nursing profession coupled with the perception of the nursing profession having unfavorable work conditions [15].

Most students reported having authoritative parents, followed by authoritarian parents, and few students reported having permissive parents. These findings are inconsistent with previous studies [28, 29]. For instance, a recent study with Qatari parents revealed that permissive parenting was the most frequently used parenting style among participants, whereas authoritarian parenting was the least used [29]. This variation in the reported parenting styles could be related to the differences in measurement tools and sample characteristics.

This study revealed that paternal authoritarian parenting was significantly associated with an increased likelihood of experiencing SAD symptoms among nursing students. This was an expected finding because authoritarian parents are more rigorous, critical, overly protective, and concerned with imposing high expectations and moral standards on their children [5]. Students raised in such a way may develop SAD and negative thoughts and feelings about their competence, impeding their professional growth. This could pose difficulties for them in clinical training as nurses are expected to make quick and effective decisions [12]. Furthermore, nurses and students with poor self-esteem may struggle to deal with stress and eventually engage in maladaptive coping strategies that further exacerbate their stress [30]. These findings emphasize the importance of implementing mental health interventions to optimize students’ learning experiences and psychological well-being. Furthermore, they support the need for programs for parents to cultivate positive parenting practices and create a supportive and caring atmosphere that enhances their psychological well-being. This finding is supported by a previous study conducted in Saudi Arabia that found that parental overprotection and criticism are significant risk factors for SAD [7]. Evidence from other countries has shown that authoritarian parenting results in a high incidence of anxiety among children, whereas authoritative parenting results in better psychological outcomes [8, 9, 11].

The study found that having an authoritative parent was significantly associated with a decreased likelihood of experiencing SAD among nursing students. Moreover, more than half of the students reported having authoritative parents. Authoritative parents are generally warm and receptive, offer encouragement and support, and foster autonomy in their children [5]. Such behaviors result in favorable psychological outcomes [10, 11, 31, 32], as demonstrated in this study. Nursing students raised in authoritative households that promote open communication and self-expression may have higher self-confidence and independence. These characteristics may help students manage the stress associated with academic and clinical training, thereby enhancing their psychological health. This finding aligns with that of a previous study conducted in China that examined 1,345 adolescents and revealed that a parental approach characterized by emotional warmth led to a reduction in SAD among adolescents [9]. However, post-hoc power analysis revealed small power, and this finding should be interpreted with caution. Accordingly, future studies with larger sample sizes are needed to confirm the association between SAD and authoritative parenting style.

The present study has several limitations that may have affected the overall findings. The use of a cross-sectional design does not reflect changes in the target group over time and cannot reveal causal relationships. While the current study had sufficient power to detect small to medium effect size, it was underpowered to detect small effect size. Accordingly, future studies with larger sample sizes are needed to confirm the findings of this study. Furthermore, the use of convenience sampling may have introduced selection bias, which limits the generalizability of the findings. The sample was recruited from a single university in Saudi Arabia, which also limits the generalizability. Moreover, students with anxiety disorders were not excluded in this study. Although the study did not collect personal data, social desirability bias could still affect the generalizability of the findings. Nevertheless, the findings are of significance, especially in the context of Saudi Arabia, as no prior studies have examined the association between SAD and parenting styles among nursing students. Furthermore, this study used valid and reliable instruments, which further strengthen its utility.

### Nursing implications

The results of this study have important implications for educators and other policymakers at nursing institutions in Saudi Arabia to work toward addressing the mental health needs of nursing students with SAD. These efforts may include early detection screenings, counseling services, and timely referrals to mental health specialists for students with SAD. Nursing educators should undergo specialized training programs to equip them with the knowledge and skills to help them recognize the signs of SAD as well as provide appropriate intervention strategies to support those affected with SAD. One effective strategy that nursing educators should consider is providing regular stress management workshops to help students, especially those with SAD, to cope with academic stress. These workshops can include techniques such as deep breathing, biofeedback, mindfulness, and the emotional freedom technique which can be conducted regularly and in collaboration with psychiatric nurses or other mental health professionals [33, 34]. Nursing educators can further provide workshops in communication skills to help students build confidence in public speaking and communicate effectively with instructors, healthcare team, and patients. Another effective strategy is a peer mentorship program in which senior students provide guidance and support to first year students [35]. Additionally, nursing educators should facilitate social skills in students with SAD which can be performed by offering social opportunities such as extracurricular activities and group projects. Nursing educators should cultivate an atmosphere where open communication, particularly regarding mental health challenges, is encouraged. This could be achieved through regular meetings with students in which they can express their feelings and concerns in a comfortable environment. Students with SAD could also benefit from additional training in nursing skills, which may help alleviate their anxiety and improve their confidence. Accordingly, nursing educators should consider providing opportunities for extra training in a safe environment where students feel comfortable in practicing and refining their nursing skills. Moreover, nursing educators and counselors must be aware of the effects of different parenting styles on students’ mental health in order to be able to work closely with the parents of students with SAD. By doing so, they can provide the necessary education to parents and optimize students’ learning experiences.

## Conclusions

This study examined the relationship between SAD and parenting styles among nursing students in Saudi Arabia. This study found that nursing students with authoritative parents were less likely to experience SAD, whereas those with authoritarian parents were more likely to experience SAD. Nursing students raised by authoritarian parents who further develop SAD can experience more challenges than other students, which may negatively influence their academic performance. Policymakers at nursing institutions should implement early detection screenings, preventive programs, and counseling services for students with SAD. Furthermore, they must design educational programs on effective parenting practices and raise awareness of the impact of authoritarian parenting on the mental health outcomes of nursing students. Future studies should continue to explore the impact of parenting practices on the development of SAD in nursing students in Saudi Arabia. As the study found an association between parenting styles and SAD in female nursing students, future research should consider investigating this association among both male and female nursing students as well as comparing the two. Research should also identify effective strategies and interventions that nursing institutions can employ to help nursing students manage their SAD symptoms.

## Data Availability

The datasets used and analyzed during the current study are available from the corresponding author on reasonable request.

## Abbreviations

SAD: Social Anxiety Disorder
PAQ: Parental Authority Questionnaire
SPIN: Social Phobia Inventory
KSAU-HS: King Saud bin Abdulaziz University for Health Sciences
